# Correlation between serum ANKRD22 and SERPING1 levels and drug resistance in pulmonary tuberculosis: A retrospective cross-sectional study

**DOI:** 10.1097/MD.0000000000045424

**Published:** 2025-10-31

**Authors:** Qianyun Zhou, Li Zhang, Mei Zhou, Lang Xiao, Niannian Liu, Jian Xu

**Affiliations:** a Department of Respiratory and Critical Care Medicine, The People’s Hospital of Yubei District of Chongqing, Chongqing, China; b Department of Infectious Diseases, The People’s Hospital of Yubei District of Chongqing, Chongqing, China.

**Keywords:** ANKRD22, drug resistance, *Mycobacterium tuberculosis*, SERPING1, tuberculosis

## Abstract

Drug-resistant tuberculosis (TB) lacks rapid blood-based biomarkers. This study examined whether serum levels of ankyrin repeat domain 22 (ANKRD22) and serpin family G member 1 (SERPING1) are associated with drug resistance in TB patients. In this retrospective cross-sectional study, 170 culture-confirmed TB patients treated from January 2023 to December 2023 were classified as drug-resistant (n = 34) or drug-susceptible (n = 136) by phenotypic drug susceptibility testing. We quantified serum ANKRD22 and SERPING1 levels by enzyme-linked immunosorbent assay, used multivariate logistic regression to identify independent risk factors, and assessed diagnostic performance with receiver operating characteristic curve analysis. Both biomarkers were significantly higher in the drug-resistant group (*P* < .001) and positively correlated with resistance (ANKRD22 *R* = 0.551, SERPING1 *R* = 0.520). Area under the curve values were 0.898 for ANKRD22, 0.875 for SERPING1, and 0.912 for combined detection. After adjustment, elevated ANKRD22 and SERPING1 remained independent predictors, along with smoking, chronic obstructive pulmonary disease, cavitary disease, previous TB exposure, and treatment interruption. Serum ANKRD22 and SERPING1 are independently associated with TB drug resistance; their combined measurement improves diagnostic accuracy and may facilitate early detection of drug-resistant TB.

## 1. Introduction

Tuberculosis (TB), a persistent infectious disease with ancient origins, continues to pose significant global public health challenges. According to reports, TB remains the second leading global cause of mortality from a single infectious agent, exceeded only by HIV/AIDS.^[1]^ The rise of drug-resistant *Mycobacterium tuberculosis (M tuberculosis*) strains, particularly multidrug-resistant tuberculosis (MDR-TB) and extensively drug-resistant tuberculosis (XDR-TB), has severely compromised TB control efforts, diminishing the efficacy of conventional therapies.^[2,3]^ Therefore, investigating the relationship between serum biomarkers and drug resistance in Mycobacterium TB may provide new insights and methods for the prevention and control of TB and the monitoring of drug resistance.

Ankyrin repeat domain 22 (ANKRD22), a nuclear-localized ankyrin repeat-containing protein, regulates gene expression through transcription factor interactions.^[4]^ Through transcription factor interactions, ANKRD22 modulates gene expression to regulate critical cellular processes including proliferation, differentiation, and apoptosis.^[5,6]^ Additionally, ANKRD22 is involved in cellular signaling pathways, and its overexpression in macrophages can promote the production of pro-inflammatory cytokines, playing a significant role in inflammatory responses.^[7]^ Therefore, in infectious diseases, changes in ANKRD22 expression levels may reflect the host’s immune response to pathogens and affect the host’s immune reaction to Mycobacterium TB.

Serpin family G member 1 (SERPING1) is a member of the serine protease inhibitor superfamily. Its molecular structure contains a typical reactive center loop that can participate in the regulation of the release of various inflammatory factors.^[8]^ A study investigating the use of SERPING1 expression levels to differentiate between active TB and latent TB infection found that SERPING1 as a biomarker for TB is heterogeneous across different regions and is associated with active TB.^[9]^ However, the role of SERPING1 in drug resistance of Mycobacterium TB remains underexplored.

This study evaluates serum ANKRD22 and SERPING1 as potential biomarkers of drug resistance through comparative analysis of expression profiles in drug-resistant versus drug-sensitive tuberculosis patients. These findings could inform more effective TB control strategies through optimized treatment regimens and containment of drug-resistant strains.

## 2. Materials and methods

### 2.1. Bioinformatics analysis

Differential expression analysis was performed using the Gene Expression Omnibus database (maintained by NCBI; https://www.ncbi.nlm.nih.gov). The microarray dataset GSE83456, containing gene expression profiles from blood samples of TB patients and healthy controls, was analyzed. Differentially expressed genes were identified with criteria of adjusted *P* < .05 and |log 2(fold change)| > 1.

### 2.2. Study subjects

Epidemiological data indicate that the prevalence of MDR-TB and XDR-TB in retreatment TB cases is 10.2% and 1.7%, respectively.^[10]^ In this study, we set the error margin not to exceed 5% (i.e., the difference between the upper and lower limits of the confidence interval is 10%) and selected a significance level of 1 ‐ α = 0.9 (2-sided test). Using the PASS 15.0 software, we calculated that at least 159 patients needed to be included to ensure the scientific validity of the study design.

**Figure 1.**
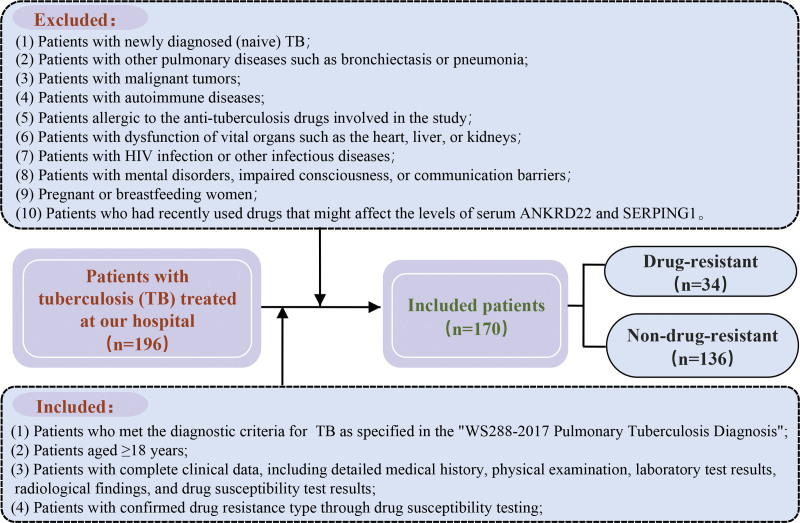
Flowchart of the screening and grouping process of TB patients. TB = tuberculosis.

We retrospectively analyzed 192 consecutive tuberculosis patients treated at our institution between January 2023 and December 2023. After applying exclusion criteria (n = 22), the final cohort comprised 170 cases (92 male, 78 female) with a mean age of 46.05 ± 5.48 years (range: 33–62 years). Figure 1 presents the flowchart of the screening and grouping process of TB patients.

Inclusion criteria: First, compliance with the diagnostic criteria for TB, this includes positive evidence of mycobacterium from sputum smear, sputum culture, or other samples, radiological features of TB lesions on chest X-ray or CT, symptoms of TB intoxication such as cough, sputum production, hemoptysis, low-grade fever, night sweats, and weight loss, a positive tuberculin purified protein derivative test, a positive interferon-γ release assay, or a positive TB antibody test. Second, age ≥ 18 years. Third, complete clinical data, including detailed medical history, physical examination, laboratory test results, radiological findings, and drug resistance test results. Fourth, patients with confirmed drug resistance type through drug susceptibility testing.

Exclusion criteria: First, new TB patients who have not been treated before. Second, patients with other pulmonary diseases such as bronchiectasis or pneumonia. Third, patients with malignant tumors. Fourth, patients with autoimmune diseases. Fifth, patients allergic to the antituberculosis drugs involved in the study. Sixth, patients with dysfunction of important organs such as heart, liver, or kidney. Seventh, patients with HIV infection or other infectious diseases. Eighth, patients with mental illness, consciousness disorders, or communication barriers. Nine, pregnant or breastfeeding women. Tenth, patients who have recently used drugs that may affect the levels of serum ANKRD22 and SERPING1.

This study was conducted in accordance with the Declaration of Helsinki. Archived serum specimens from the hospital biobank were used; all samples were deidentified and linked to clinical data by a coded study number only. The study protocol was approved by the Ethics Committee of The People’s Hospital of Chongqing Yubei District (Approval No. 2023SA01), which also waived the requirement for re-consent due to minimal risk and impracticability of recontact.

### 2.3. Collection of clinical data

General information of the patients was collected, including gender, age, BMI, history of smoking and alcohol consumption, region of residence, hypertension, diabetes, coronary heart disease, chronic obstructive pulmonary disease (COPD), presence of lung cavities, history of TB exposure, and whether the treatment process was interrupted.

### 2.4. Detection of serum ANKRD22 and SERPING1 levels

Serum ANKRD22 and SERPING1 levels were measured by enzyme-linked immunosorbent assay (ELISA) following manufacturer instructions. All tests were conducted using ELISA kits provided by Shanghai Kbio Biotechnology (product codes: CB16975-Hu for ANKRD22 and CB15120-Hu for SERPING1). Serum samples collected at admission were processed within 2 hours: clotted at room temperature (15 ± 5 minutes), centrifuged at 2500 ± 500 rpm for 20 minutes, and stored at −80°C. ELISA analysis included 60 minutes incubation at 37°C with standard curve samples processed identically to test specimens. After washing, the chromogen was added for color development in the dark for 15 minutes. The reaction was stopped, and the optical density was determined at 450 nm via a microplate reader. Concentrations were then derived from a standard curve.

### 2.5. Drug susceptibility testing

All enrolled TB patients in this study provided sputum samples in the morning using the natural cough method: Patients first rinsed their mouth with lukewarm water to remove oral and pharyngeal secretions (by making the sound “ha” to clear the throat, rather than coughing), and then forcefully coughed up sputum from the deep bronchial region. The sputum volume should be no <1 mL and was expectorated into a sterile sputum container, which was then closed. Before sample collection, patients were clearly instructed that the specimen should be from the lower respiratory tract, not from the upper respiratory tract’s nasal or pharyngeal secretions.

The drug susceptibility of *M tuberculosis* was evaluated using the solid medium proportion method in phenotypic drug susceptibility testing. This method included 4 first-line antituberculosis drugs (isoniazid, rifampicin, streptomycin, and ethambutol) and second-line drugs (fluoroquinolones, bedaquiline, and linezolid).

### 2.6. Definitions of drug resistance

Drug resistance patterns in this study were classified according to the WHO definitions:

MDR-TB: Resistance to at least isoniazid and rifampicin.

XDR-TB: MDR-TB strains with additional resistance to any fluoroquinolone (e.g., levofloxacin, moxifloxacin) and at least 1 group A second-line drug (e.g., bedaquiline, linezolid).^[11–13]^

In this study, patients with MDR-TB or XDR-TB were grouped as drug-resistant (n = 34), while those fully susceptible to all tested drugs were grouped as nondrug-resistant (n = 136).

### 2.7. Statistical analysis

Statistical analyses were conducted using SPSS 26.0 (IBM, Armonk, NY) and GraphPad Prism 9.5. Continuous data normality was assessed via the Kolmogorov–Smirnov test. Normally distributed data were presented as mean ± standard deviation ($\bar{x}\pm s$) and compared using the *t* test; non-normally distributed data were presented as median and interquartile range [*M (P25, P75*)] and compared using the Mann–Whitney *U* test. Categorical data were described as counts and percentages (%) and compared using the chi-square (*χ*^2^) test. The correlation between serum ANKRD22 and SERPING1 levels in TB patients was analyzed using Spearman correlation. Receiver operating characteristic curves were generated to evaluate the predictive performance of ANKRD22 and SERPING1 for drug resistance. Multivariate logistic regression (MLR) analysis was used to identify risk factors for drug resistance. A *P*-value < .05 was considered statistically significant.

## 3. Results

### 3.1. Results of bioinformatics analysis

**Figure 2.**
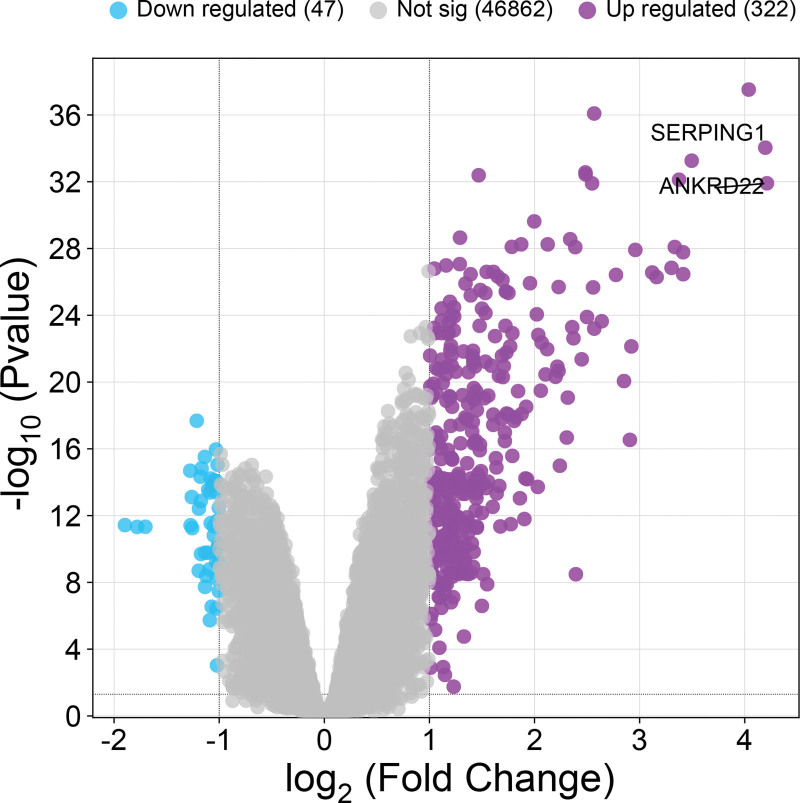
Results of GSE83456 microarray analysis.

Analysis of the GSE83456 dataset demonstrated significant upregulation of both ANKRD22 and SERPING1 in peripheral blood from TB patients compared to healthy controls (Fig. 2).

### 3.2. Comparison of general data of TB patients

Our study analyzed 170 tuberculosis patients, identifying 34 drug-resistant cases. As detailed in Table 1, the drug-resistant group showed significantly higher prevalence of: smoking history (*P* < .05), COPD (*P* < .05), pulmonary cavities (*P* < .05), documented TB exposure (*P* < .05), and treatment interruption (*P* < .05) compared to drug-sensitive patients.

**Table 1:** Comparison of demographic and clinical parameters of TB patients.

Baseline characteristics	Drug-resistant group (n = 34)	Nondrug-resistant group (n = 136)	*t*/*χ*^2^	*P*
Gender/n (%)			0.024	.878
Male	18 (52.94)	74 (54.41)		
Female	16 (47.06)	62 (45.59)		
Age ($\bar{x}\pm s$, yr)	45.47 ± 5.17	46.20 ± 5.57	-0.691	.490
BMI ($\bar{x}\pm s$, kg/m^‐2^)	22.96 ± 2.73	23.18 ± 2.25	-0.434	.666
Smoking/n (%)	26 (76.47)	51 (37.50)	16.671	<.001
Alcohol consumption/n (%)	20 (58.82)	66 (48.53)	1.153	.283
Residence area/n (%)			0.848	.357
Urban	19 (55.88)	64 (47.06)		
Rural	15 (44.12)	72 (52.94)		
Hypertension/n (%)	11 (32.35)	54 (39.71)	0.623	.430
Diabetes/n (%)	12 (35.29)	50 (36.76)	0.025	.873
Coronary heart disease/n (%)	19 (55.88)	79 (58.09)	0.054	.816
COPD/n (%)	27 (79.41)	59 (43.38)	14.125	<.001
Lung cavities/n (%)			11.987	.001
Yes	22 (64.71)	44 (32.35)		
No	12 (35.29)	92 (67.65)		
Tuberculosis exposure history/n (%)			29.423	<.001
Yes	20 (58.82)	20 (14.71)		
No	14 (41.18)	116 (85.29)		
Treatment interruption/n (%)			13.570	<.001
Yes	26 (76.47)	56 (41.18)		
No	8 (23.53)	80 (58.82)		

COPD = chronic obstructive pulmonary disease, TB = tuberculosis.

### 3.3. Comparison of serum ANKRD22 and SERPING1 levels in TB patients with different resistance status

**Figure 3.**
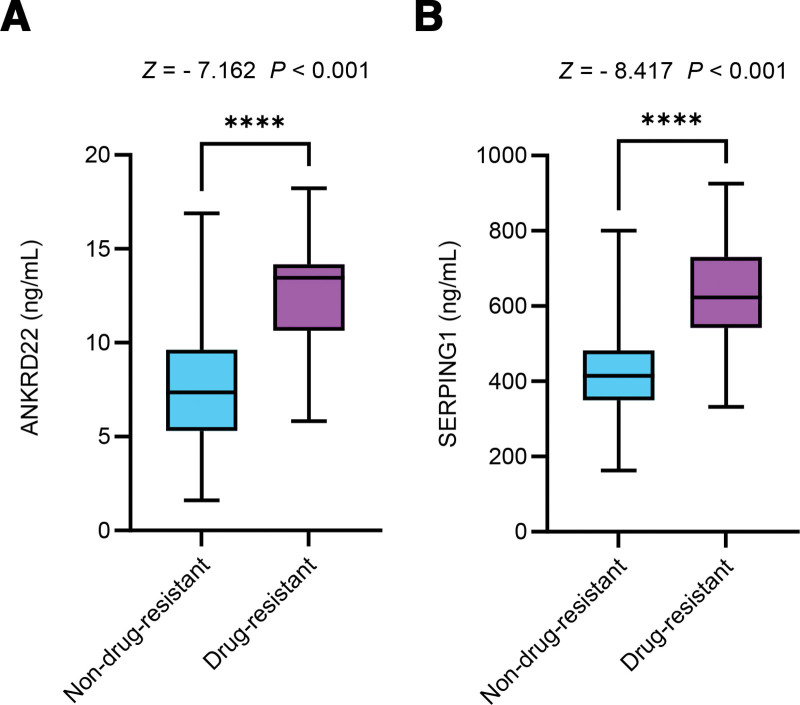
Comparison of serum ANKRD22 and SERPING1 levels in TB patients with different resistance status. ANKRD22 = ankyrin repeat domain 22, SERPING1 = serpin family G member 1, TB = tuberculosis.

Drug-resistant patients exhibited significantly elevated serum ANKRD22 and SERPING1 levels compared to drug-sensitive controls (both *P* < .001; Fig. 3).

### 3.4. Relationship between serum ANKRD22, SERPING1 levels and drug resistance

**Figure 4.**
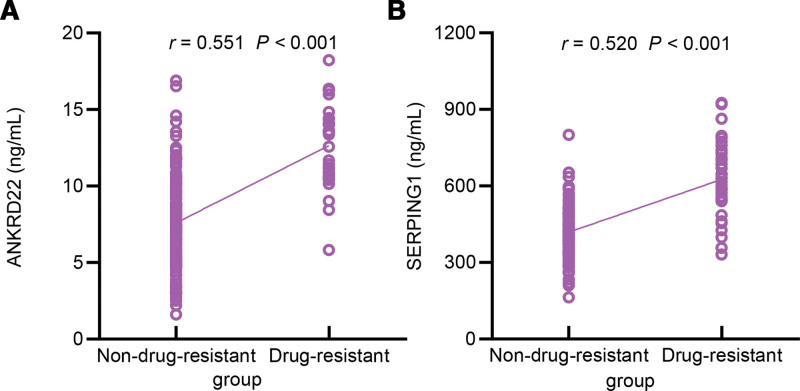
Relationship between serum ANKRD22, SERPING1 levels and drug resistance. ANKRD22 = ankyrin repeat domain 22, SERPING1 = serpin family G member 1.

Serum ANKRD22 (*R* = 0.551) and SERPING1 (*R* = 0.520) concentrations both showed significant positive correlations with drug resistance status (both *P* < .001) (Fig. 4).

### 3.5. Correlation between serum ANKRD22 and SERPING1 levels in the drug-resistant group

**Figure 5.**
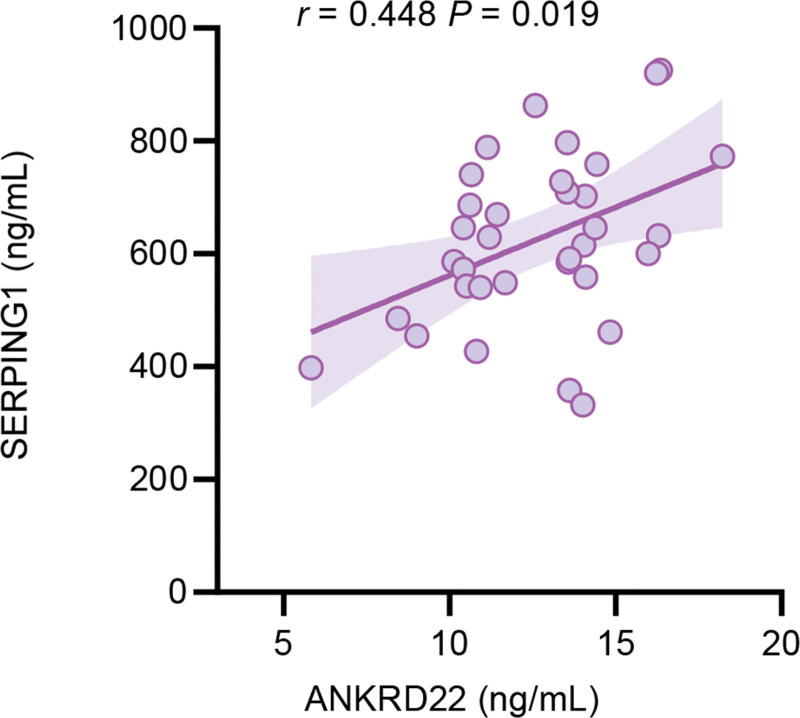
Correlation between serum ANKRD22 and SERPING1 levels in the drug-resistant group. ANKRD22 = ankyrin repeat domain 22, SERPING1 = serpin family G member 1.

Spearman correlation analysis showed that serum ANKRD22 and SERPING1 levels were positively correlated in drug-resistant TB patients (*R* = 0.448, *P* = .019) (Fig. 5).

### 3.6. Predictive value of serum ANKRD22 and SERPING1 levels for drug resistance in TB patients

**Figure 6.**
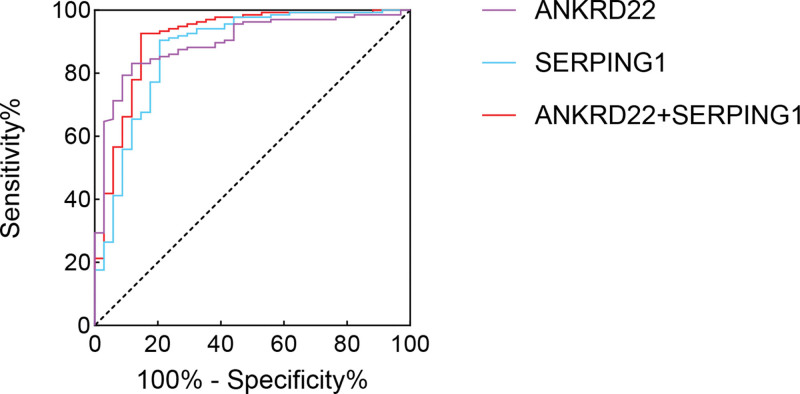
ROC curves for predicting drug resistance in TB patients based on serum ANKRD22 and SERPING1 levels. ANKRD22 = ankyrin repeat domain 22, SERPING1 = serpin family G member 1, TB = tuberculosis.

Receiver operating characteristic analysis demonstrated excellent predictive accuracy for drug resistance, with area under the curve (AUC) values of 0.898 (ANKRD22) and 0.875 (SERPING1). The combined detection of ANKRD22 and SERPING1 yielded an AUC of 0.912, which was superior to the individual detection of either biomarker, as shown in Table 2 and Figure 6.

**Table 2:** Predictive value of serum ANKRD22 and SERPING1 levels for drug resistance in TB patients.

Variable	cutoff	Sensitivity%	Specificity%	AUC	95% CI
ANKRD22	10.36 ng/mL	83.09	88.24	0.898	0.843–0.952
SERPING1	535.00 ng/mL	90.44	79.41	0.875	0.800–0.951
ANKRD22 + SERPING1	–	92.65	85.29	0.912	0.850–0.975

ANKRD22 = ankyrin repeat domain 22, AUC = area under the curve, SERPING1 = serpin family G member 1, TB = tuberculosis.

### 3.7. MLR analysis of factors affecting drug resistance in TB patients

**Figure 7.**
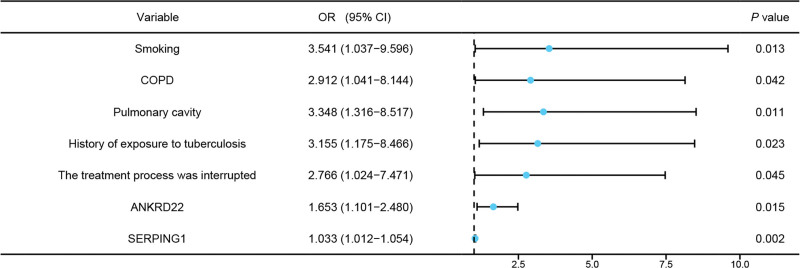
Forest plot of MLR analysis for factors affecting drug resistance in TB patients. TB = tuberculosis.

MLR analysis was performed with drug resistance in TB patients as the dependent variable (yes = 1, no = 0) and the following variables as independent variables: smoking status (yes = 1, no = 0), presence of COPD (yes = 1, no = 0), presence of lung cavities (yes = 1, no = 0), history of TB exposure (yes = 1, no = 0), treatment interruption (yes = 1, no = 0), and serum levels of ANKRD22 and SERPING1. Six parameters emerged as significant predictors of TB drug resistance: smoking status, COPD comorbidity, lung cavitation, TB contact history, treatment interruption, and elevated ANKRD22/SERPING1 biomarker levels (complete statistical analysis in Table 3 and Fig. 7).

**Table 3:** MLR analysis of factors affecting drug resistance in TB patients.

Variable	β	SE	Walds	*P*	OR (95% CI)
Smoking	1.265	0.509	6.182	.013	3.541 (1.037–9.596)
COPD	1.069	0.525	4.150	.042	2.912 (1.041–8.144)
Pulmonary cavity	1.208	0.476	6.435	.011	3.348 (1.316–8.517)
History of exposure to tuberculosis	1.149	0.504	5.203	.023	3.155 (1.175–8.466)
The treatment process was interrupted	1.018	0.507	4.029	.045	2.766 (1.024–7.471)
ANKRD22	0.502	0.207	5.882	.015	1.653 (1.101–2.480)
SERPING1	0.032	0.010	9.466	.002	1.033 (1.012–1.054)

ANKRD22 = ankyrin repeat domain 22, COPD = chronic obstructive pulmonary disease, MLR = multivariate logistic regression, SERPING1 = serpin family G member 1, TB = tuberculosis.

## 4. Discussion

TB maintains its position as a global health priority, with antimicrobial resistance patterns—especially MDR-TB and XDR-TB strains—creating significant barriers to effective disease management and control.^[14]^ This study characterizes the diagnostic potential of serum ANKRD22 and SERPING1 as biomarkers for drug-resistant TB, with implications for timely therapeutic decision-making.

Bioanalysis demonstrated ANKRD22 and SERPING1 upregulation in TB. Their serum levels were elevated in drug-resistant cases (*P* < .001) and correlated with resistance status (*R *= 0.551 and 0.520 respectively, *P* < .001). MLR supported their risk factor status, indicating potential involvement in drug-resistant TB.

ANKRD22 could potentially drive drug resistance through: regulation of host cell cycle genes favoring mycobacterial survival; and modulation of macrophage activation states that dampens inflammatory responses and impairs pathogen clearance.^[15]^ SERPING1 may support drug resistance by modulating host immunity, potentially through suppression of inflammatory responses that would normally eliminate resistant bacterial populations.^[16,17]^

Significant correlation was observed between serum ANKRD22 and SERPING1 levels. Both biomarkers demonstrated high predictive value for drug resistance when assessed independently (ANKRD22: AUC 0.898; SERPING1: AUC 0.875). The combined detection of these 2 biomarkers yielded an AUC of 0.912, which was superior to individual testing. The simultaneous assessment of ANKRD22 and SERPING1 expression demonstrates enhanced predictive capacity for identifying drug resistance, thereby enabling earlier detection and therapeutic intervention in drug-resistant TB. These results provide substantial evidence to support the clinical application of this biomarker combination in the preliminary screening of drug-resistant TB cases. The dual-marker approach involving ANKRD22 and SERPING1 shows promising clinical utility as a potential predictor of tuberculosis drug resistance, offering novel perspectives for improving diagnostic timelines and treatment strategies in drug-resistant TB management.

Research has demonstrated that smoking increases the body’s resistance to antituberculosis drugs, leading to prolonged treatment duration, increased treatment costs, and a higher incidence of adverse reactions. It is thus one of the significant risk factors for drug resistance in TB.^[18–20]^ Additionally, smoking may impair the host’s ability to clear *M tuberculosis* by affecting pulmonary immune function and inflammatory responses, thereby promoting the emergence of drug-resistant strains.^[21]^

Patients with COPD have impaired pulmonary immune function and increased inflammatory responses,^[22]^ which facilitate the colonization and proliferation of *M tuberculosis* in the lungs and increase the risk of drug-resistant strains emerging. Existing evidence indicates that the hypoxic microenvironment and immunologically protected nature of pulmonary cavities create favorable conditions for accelerated *M tuberculosis* proliferation and the emergence of drug-resistant mutations.^[23]^ Moreover, the poor drug penetration within cavities further increases the selective pressure for drug-resistant strains, leading to the occurrence of drug resistance.^[24]^

Patients with prior tuberculosis exposure exhibit an increased risk of developing drug-resistant TB, which may result from either primary infection with resistant *M tuberculosis* strains or the progressive acquisition of resistance during prolonged infection.^[25]^ Studies by Tola HH et al have demonstrated that treatment interruption constitutes a major risk factor for unfavorable therapeutic outcomes and the development of further drug resistance in tuberculosis cases.^[26]^ MLR analysis in this investigation substantiated the statistically significant correlation between these identified factors and TB drug resistance.

These results offer clinically relevant evidence to support early detection and therapeutic management of drug-resistant TB, potentially facilitating treatment optimization and containment of resistant strains.

While this study yields significant findings, certain limitations warrant consideration. The restricted sample size and single-institution design may affect the external validity of the results. Subsequent research should incorporate larger, multicenter cohorts to verify the broader clinical utility of these biomarkers. Additionally, although the investigation established correlations between serum ANKRD22/SERPING1 levels and drug resistance, the precise molecular mechanisms remain to be elucidated. Future research could employ multi-omics approaches, including gene expression and protein interaction studies, to further elucidate the specific roles of these biomarkers in drug resistance. Additionally, exploring other potential biomarkers could contribute to the development of a more comprehensive predictive model for drug resistance.

This investigation demonstrates a significant association between serum ANKRD22/SERPING1 expression profiles and TB drug resistance. The combined assessment of both biomarkers showed enhanced predictive performance compared to individual marker analysis. These findings identify novel diagnostic targets for early detection of drug-resistant TB, potentially informing clinical decision-making and therapeutic strategies. Further validation of these biomarkers’ clinical applicability and mechanistic studies elucidating their role in resistance development are warranted.

## Author contributions

**Conceptualization:** Qianyun Zhou.

**Data curation:** Qianyun Zhou, Li Zhang.

**Formal analysis:** Li Zhang, Mei Zhou.

**Funding acquisition:** Lang Xiao, Niannian Liu.

**Investigation:** Li Zhang, Niannian Liu, Jian Xu.

**Methodology:** Lang Xiao, Jian Xu.

**Project administration:** Qianyun Zhou, Li Zhang.

**Resources:** Mei Zhou, Niannian Liu.

**Software:** Qianyun Zhou, Jian Xu.

**Supervision:** Qianyun Zhou.

**Validation:** Li Zhang.

**Visualization:** Lang Xiao.

**Writing – original draft:** Li Zhang, Niannian Liu, Jian Xu.

**Writing – review & editing:** Qianyun Zhou, Mei Zhou, Lang Xiao
